# Effects of two peroxide enzymatic denture cleaners on *Candida albicans* biofilms and denture surface

**DOI:** 10.1186/s12903-020-01176-6

**Published:** 2020-07-08

**Authors:** Ying Han, Xiaodan Liu, Yu Cai

**Affiliations:** 1 Department of Oral Medicine, Peking University School and Hospital of Stomatology & National Clinical Research Center for Oral Diseases & National Engineering Laboratory for Digital and Material Technology of Stomatology & Beijing Key Laboratory of Digital Stomatology, Beijing, China; 2 Department of Stomatology, Peking University Third Hospital, Beijing, China; 3 Department of Periodontology, Peking University School and Hospital of Stomatology & National Clinical Research Center for Oral Diseases & National Engineering Laboratory for Digital and Material Technology of Stomatology & Beijing Key Laboratory of Digital Stomatology, 22 Zhongguancun South Avenue, Haidian District, Beijing, 100081 PR China; 4 Central Laboratory, Peking University School and Hospital of Stomatology & National Clinical Research Center for Oral Diseases & National Engineering Laboratory for Digital and Material Technology of Stomatology & Beijing Key Laboratory of Digital Stomatology, 22 Zhongguancun South Avenue, Haidian District, Beijing, 100081 PR China

## Abstract

**Objective:**

To compare the antifungal action of two commercially available denture cleaning agents to that of standard clinical solutions, and determine their effects on the polymethyl methacrylate (PMMA) acrylic resin denture surface.

**Methods:**

*Candida albicans* growth was analyzed by colony forming assay, and the methyl thiazolyl tetrazolium (MTT) assay was used to evaluate biofilm formation and cell adhesion. The morphology and roughness of PMMA acrylic resin surface was measured by scanning electron microscopy (SEM) images and stylus method.

**Results:**

Clene®, Polident® and 3% NaHCO_3_ solutions showed significantly greater antifungal effects in terms of both inhibiting growth and biofilm formation. In addition, Clene® solution prevented adhesion of *C. albicans* on cell culture plates compared to filter-sterile tap water, whereas other reagents did not have an inhibitory effect. One-month immersion in the different cleaning reagents significantly inhibited fungal adhesion on the PMMA surface Clene®, Polident® and 3% NaHCO_3_ showed greater effect compared to PBS and filter-sterile tap water. Finally, none of the cleansing agents significantly affected the morphology and roughness of the PMMA surface.

**Conclusion:**

Clene®, Polident® and 3% NaHCO_3_ solutions can inhibit *C. albicans* growth and biofilm formation to some extent on cell culture plates, and significantly inhibit fungal adhesion on the PMMA surface without affecting surface morphology and roughness.

## Introduction

Denture-induced stomatitis (DS) is caused by the opportunistic yeast *Candida spp.*, and is characterized by white pseudomembranous lesions covering large areas of the oral mucosa such as tongue or palate [1]. *C. albicans* is one of the major causative organism in *Candida spp.*, primarily due to its ability to rapidly adhere to, and form resilient drug-resistant biofilms on the soft and hard oral cavity tissues [2]. *C. albicans* is a dimorphic yeast that can exist in both hyphal and yeast forms depending on the environment [3]. In addition, *C. albicans* is also the most common *Candida* species found in the healthy human oral mucosa, and causes candidiasis due to its adherence properties and pathogenicity [4]. The transition of resident *Candida* from the commensal to pathogenic form depends on various local and systemic factors*.*

Poorly fitted dentures occlude the oral mucosa and inhibit salivary flow, leading to *Candida* overgrowth [3, 5]. In addition, the denture base materials are an ideal surface for plaque and calculus formation, which further promote *C. albicans* adherence and growth. Compared to other dental materials, polymethyl methacrylate (PMMA) acrylic resin is more frequently colonized by this yeast [6], and usually requires periodic cleaning by chemicals [7] like alkaline peroxides, alkaline hypochlorite, acids, disinfectant and enzymes [8] to inhibit biofilm growth. The most commonly-used denture cleansers are the immersion type which clean as well as decontaminate the prostheses [9]. Polident® is a peroxide- and enzyme-based cleaning agent with considerable antifungal effects [9, 10]. Clene® is similar to Polident® in composition and can effectively inhibit *Candida* growth. The aim of this study was to compare the above denture cleansers in terms of their effects on *C. albicans* adherence and biofilm formation, and on the mechanical characteristics of PMMA acrylic resin.

## Materials and methods

### Preparation of test solutions

Solutions of Clene® (Bitec global group, Japan), Polident® (PTI Royston LLC, USA), 3% sodium bicarbonate (NaHCO_3_, Tianjin Lisheng Pharmaceutical Co. LTD, China) and phosphate-buffer Saline (PBS, Gibco® Invitrogen™, USA) were prepared at the recommended concentrations by adding one tablet or sachet in 200 ml filter-sterile tap water at room temperature (RT).

### *C. albicans* culture

*C. albicans* strain ATCC 90028 (School of Stomatology, Peking University, China) was cultured in Roswell Park Memorial Institute (RPMI) 1640 medium (HyClone, Logan, USA) at 37 °C for 48 h, and the primary culture was passaged and grown for another 24 h. The yeast cells were harvested by centrifuging the broth cultures (Labofuge 400 R, Function-line, Heraeus Instruments, Bensheim, Germany) at 3000 rpm for 10 min. The cell pellets were washed twice with PBS, and re-suspended at the final density of 10^5^ CFU/ml for evaluating antifungal and anti-adhesion effects on the PMMA acrylic resin base [11], and 10^6^ CFU/ml for evaluating anti-biofilm and anti-adhesion effects on cell culture plates [12].

### Evaluation of antifungal effects

One milliliter of the *C. albicans* suspension was dispensed in five separate sterile test tubes each, and respectively treated with 9 ml Clene®, Polident®, 3% NaHCO_3_, PBS and filter-sterile tap water for 30, 60 and 120 min. The tubes were sonicated at 7-W for 30s [13] and vortexed at the stipulated time points, and 1 ml aliquots were plated on Sabourad dextrose agar (SDA, Antobio Co., China) using a spiral plater (Spiral system; Interscience, Saint-Nom-La-Breeches, France). The plates were incubated at 37 °C for 24 h, and the number of colonies were counted. Three biological replicates with three technical replicates each were tested for every condition.

### Evaluation of anti-biofilm effects

One hundred microliter aliquots of *C. albicans* suspension (10^6^ CFU/ml) were seeded in pre-sterilized 96-well cell culture plates (Corning Co., USA), and incubated for 90 min (adhesion phase) at 37 °C in an orbital shaker at 75 rpm [14]. The non-adherent cells were removed by gently washing thrice with PBS, and 100 μl RPMI-1640 was added to each well. After incubating for another 24 h, the medium was removed and the wells were washed twice with PBS, followed by addition of 100 μl each of fresh RPMI and a cleansing solution. The cells were incubated for 48 h [15] and the non-adherent cells were removed. The resultant biofilm was quantified using methyl thiazolyl tetrazolium (MTT) assay (Sigma Chemical Co., USA). Briefly, 20 μl MTT (5 mg/ml in PBS) was added to each well, and incubated for 4 h in the dark. The supernatant was aspirated and 100 μl dimethylsulfoxide (DMSO, Sigma Chemical Co., USA) was added to each well, and the optical density (OD) was measured at 490 nm after 30 mins. The experiment was repeated at least three times. Each experiment was set up in triplicate with three replicates per sample.

### Evaluation of anti-adhesion effects

*C. albicans* suspension (10^6^ CFU/ml) was seeded in 96-well cell culture plate, and incubated with 200 μl of the different solutions for 90 mins (adhesion phase) at 37 °C in an orbital shaker at 75 rpm. After gently washing thrice with PBS to remove the non-adherent cells, the biofilm was quantified by MTT assay as described above. All experiments were performed in duplication of three independent sessions.

### Evaluation of microbial adhesion on PMMA base

Forty-five circular heat-cured PMMA resin Vertex Rapid Simplified (Vertex-Dental B.V.: Zeist, Netherlands) plates (diameter 10 mm and thickness 2 mm) were fabricated, and sanded with 360, 400 and 600-grit abrasive papers (Carbimet, Buehler, Lake Bluff, IL, USA) under refrigeration to remove the scratches formed during grinding. After buffing with a polishing cloth and 1 μm diamond suspension (Metadi diamond suspension, Buehler, Lake Bluff, IL, USA), the disks were sonicated in filter-sterile tap water in a 900-W microwave oven for 3 mins at 360-W, and for another 3 mins at 810-W after a 4 mins interval [14]. The disks were then washed with a high-pressure water spray to eliminate any surface contaminants, and finally sterilized with 95% ethanol. The prepared disks were first immersed in artificial saliva [15] (0.02% calcium chloride, 0.11% sodium phosphate, 0.17% sodium bicarbonate and 0.2% sodium azide) for 24 h at 37 °C, and then transferred to 40 ml of the respective cleansing solution/filter-sterile tap water. After a 4-week incubation with daily change of solution, the disks were taken out, washed with PBS, and immersed in filter-sterile tap water for 24 h at 37 °C [9]. To measure *C. albicans* adhesion, 20 ml *C. albicans* suspension (10^5^ CFU/ml) was added to each tube and incubated for 90 mins. After washing with PBS to remove non-adherent cells, the disks were transferred to polypropylene tubes containing 3 ml sterile PBS each, and sonicated at 7-W for 30s [13]. The resulting single-cell suspension was plated on SDA, cultured at 37 °C for 24 h, and the number of colonies were counted by using a spiral plater. All experiments were performed with three independent sections on three independent sessions.

### Evaluation of the surface morphology and roughness of PMMA base

Forty-five circular heat-cured PMMA base disks were fabricated as described above, immersed in artificial saliva for 24 h at 37 °C, and then in 40 ml of the respective cleansing solution/ filter-sterile tap water for 8 h. The disks were removed and air dried, and the surface morphology micropatterns were analyzed by scanning electron microscopy (SEM). The roughness of the surface was measured in 3 different areas by the stylus method (Surftest SJ-400, Mitutoyo Industry, Japan), and the mean roughness was calculated. All experiments were performed with three independent sections on three independent sessions.

### Statistical analysis

All results are expressed as the mean ± standard deviation (SD). Data were analyzed using SPSS 13.0 software (IBM, Chicago, USA), and compared using one-way analysis of variance (ANOVA) followed by pair-wise Bonferroni test. *P* < 0.05 was considered statistically significant.

## Results

### Growth of *C. albicans*

**Fig. 1:**
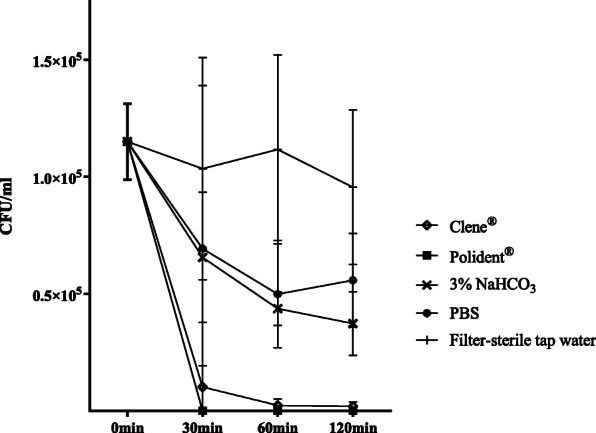
The mean ± SD numbers of colony-forming units (CFUs) after 30, 60 and 120 mins of incubation with the different cleansers

All denture cleansers decreased the growth of *C. albicans*, evaluated in terms of the number of colonies, in a time-dependent manner compared to filter-sterile tap water (Fig. 1 and Table 1). The inhibitory effects of Clene® and Polident® were significantly higher compared to that of 3% NaHCO_3_ (*P* < 0.05) and PBS (*P* < 0.05), while no significant differences were observed between the two commercial cleansers, or between 3% NaHCO_3_ and PBS. As shown in the Table 1, Clene®and Polident® decreased fungal growth by approximately 98 and 100% respectively, while 3% NaHCO_3_ and PBS achieved only 67.54 and 51.30% reduction.

**Table 1:** Mean and standard deviation (SD) in number (10^5^CFU/ml) of *Candida spp.* over the evaluation periods of 30, 60 and 120 min (*n* = 9)

Solutions		Mean (SD) (10^5^CFU/ml)			Percent (%)
	0 min(all)	30 min	60 min	120 min	(0 min–120 min)/0 min
Clene®	1.15 (0.16)	0.10 (0.09)	0.02 (0.03)	0.02 (0.02)	98.27
Polident®		0.00 (0.00)	0.00 (0.00)	0.00 (0.00)	100
3% NaHCO_3_		0.66 (0.28)	0.44 (0.07)	0.37 (0.14)	67.54
PBS		0.69 (0.70)	0.50 (0.23)	0.56 (0.20)	51.30
Filter-sterile tap water		1.03 (0.47)	1.12 (0.40)	0.96 (0.33)	16.52

### Biofilm formation

**Fig. 2:**
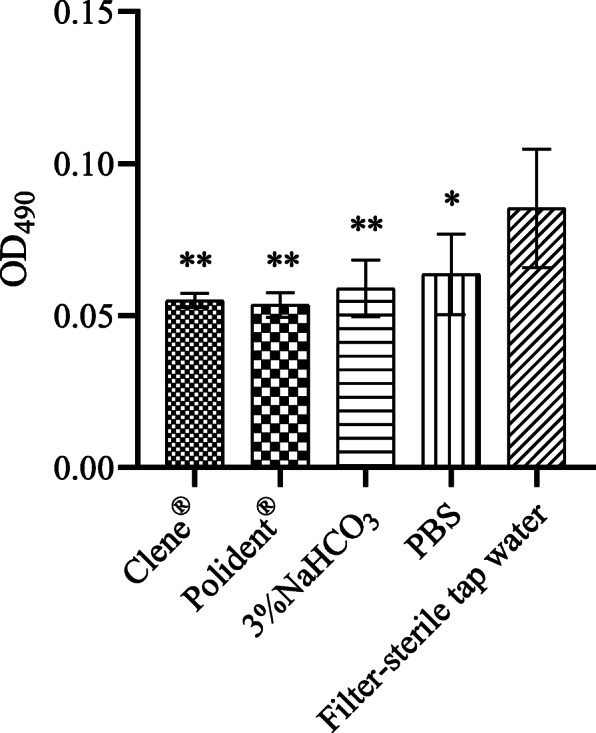
ODs indicating biofilm formation after different treatments. The data represent the mean ± SD from three independent experiments of each sample. *P < 0.05, **P < 0.01 compared with the filter-sterile tap water group

Compared to filter-sterile tap water (OD_490_ = 0.09 ± 0.02), all reagents inhibited *C. albicans* biofilm formation to some extent. However, no significant differences were detected among Clene® (OD_490_ = 0.06 ± 0.00), Polident® (OD_490_ = 0.05 ± 0.00), 3% NaHCO_3_ (OD_490_ = 0.06 ± 0.01), and PBS (OD_490_ = 0.06 ± 0.01) (Fig. 2).

### *C. albicans* adhesion on tissue culture-treated polystyrene substrate

**Fig. 3:**
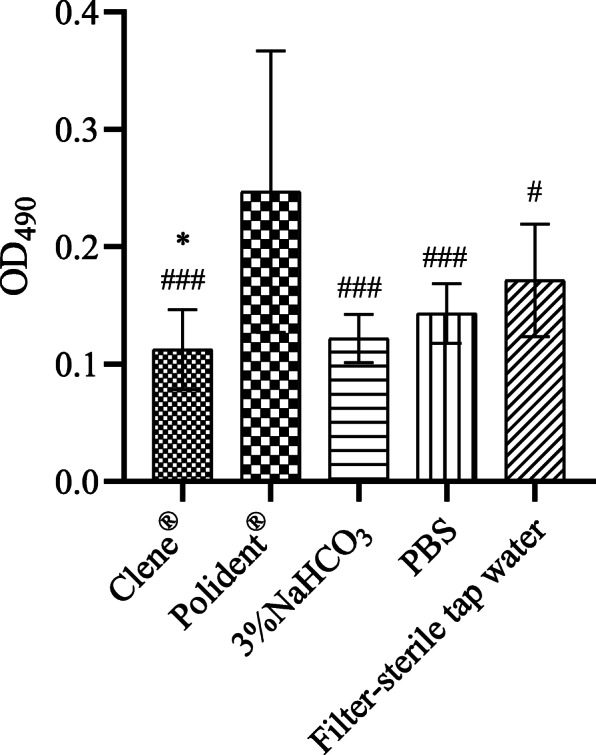
ODs indicating cell adhesion after different treatments. The data represent the mean ± SD from three independent experiments of each sample. *P < 0.05 compared with the filter-sterile tap water group. ^#^*P* < 0.05, ^###^*P* < 0.001 compared with the Polident® group

Clene® (OD_490_ = 0.11 ± 0.03) prevented adhesion of *C. albicans* compared to filter-sterile tap water (OD_490_ = 0.17 ± 0.05). Interestingly, Polident® (OD_490_ = 0.25 ± 0.12) promoted adhesion of *C. albicans*, resulting in dense cellular layers compared to that seen in the Clene®, 3% NaHCO_3_ (OD_490_ = 0.12 ± 0.02), PBS (OD_490_ = 0.14 ± 0.03) and filter-sterile tap water (*P* < 0.05) groups. Compared to PBS and filter-sterile tap water group, 3% NaHCO_3_ did not significantly inhibit the adhesion of *C. albicans*. Furthermore, no significant differences were seen between Clene® and 3% NaHCO_3_ (Fig. 3).

### *C. albicans* adhesion on PMMA base

**Fig. 4:**
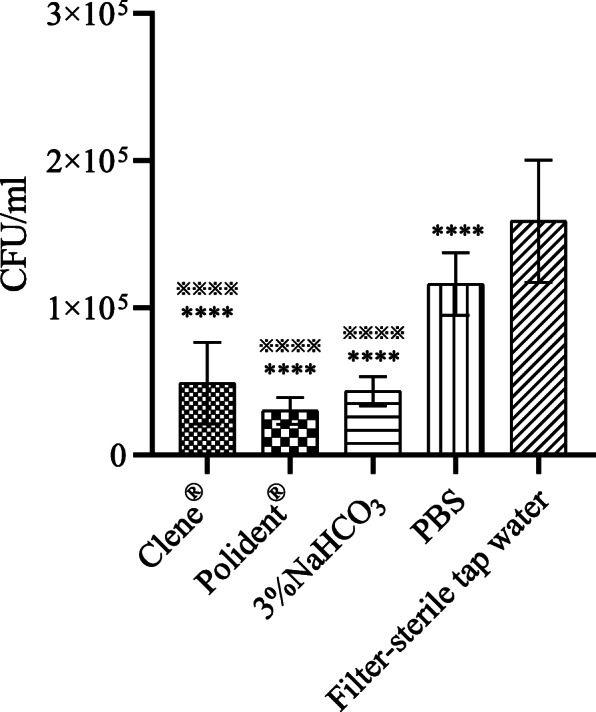
* C. albicans* colony counts after different treatments. The data show the mean ± SD from three independent experiments of each sample. ****P < 0.0001 compared with the filter-sterile tap water group; ^※※※※^*P* < 0.0001 compared with the PBS group

Compared to PBS and filter-sterile tap water, Clene®, Polident® and 3% NaHCO_3_ significantly reduced *C. albicans* adhesion on the PMMA base surface (*P <* 0.05) (Fig. 4).

### Surface morphology and roughness of PMMA base

**Fig. 5:**
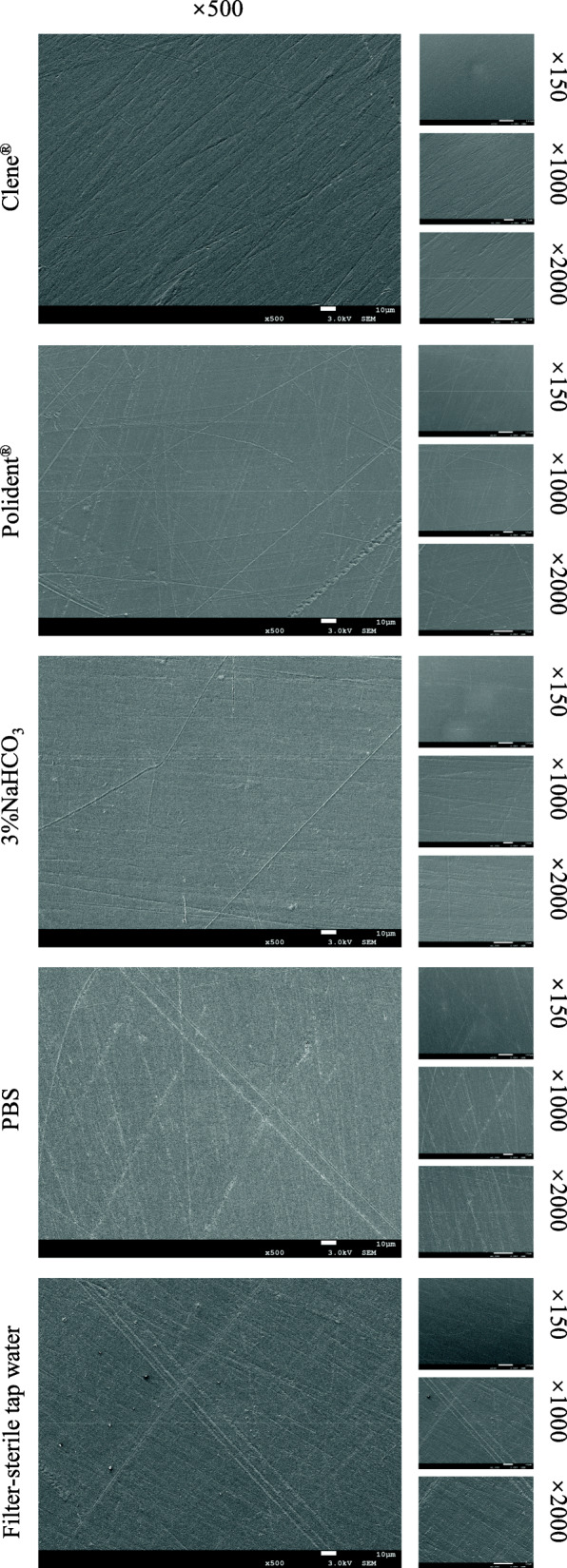
Scanning electron microscopic visualizations at 4 magnifications (×150, ×500, ×1000, ×2000) of the surface morphology and roughness of PMMA surface which treated overnight with Clene®, Polident®, 3%NaHCO_3_, PBS and filter-sterile tap water

The surface roughness of the PMMA base disks was not significantly affected even after overnight immersion in the different cleansing reagents (Table 2), and no significant differences were observed between the different groups on PMMA base surface morphology (Fig. 5).

**Table 2:** Mean and standard deviation (SD) for the Surface Roughness (Ra, μm) of the PMMA Base for solutions (*n* = 9)

Solutions	Mean (SD)
Clene®	0.12 (0.03)
Polident®	0.12 (0.04)
3% NaHCO_3_	0.12 (0.04)
PBS	0.13 (0.03)
Filter-sterile tap water	0.13 (0.03)

Data indicate no statistically significant difference (*P* > 0.05)

## Discussion

Denture stomatitis (DS) is a frequent complication in patients with dentures, especially in the elderly with limited salivary flow. Poorly fitted dentures occlude the oral mucosa and inhibit salivary flow, leading to *Candida albicans* overgrowth [3, 5]. Furthermore, accumulation of plaque and calculus on the denture base materials encourage the adherence and growth of *C. albicans*. Compared to other dental materials, PMMA acrylic resin is more frequently colonized by this yeast [6], and the resulting biofilm has to be removed periodically by chemical and physical methods [7]. Several chemical denture cleansers have been formulated that can control and even eradicate fungal infection from the denture surface [8].

Polident® is a commercial chemical denture cleanser that can significantly inhibit the proliferation of *C. albicans* [9, 10, 15, 16]. In addition, studies also show that sodium bicarbonate can effectively inhibit and disperse yeast biofilms [17, 18]. Clene® has a similar composition as Polident® but its effect on *C. albicans* growth and the PMMA acrylic resin denture surface is not well known. Compared to 3% NaHCO_3_, we found both Clene® and Polident® significantly inhibited the growth of *C. albicans* in a time-dependent manner. Ufuk et al. also showed that Polident® reduced 26 and 50% of the plaques within 30 and 60 mins of immersion respectively [9], while Ghalichebaf et al. reported < 30% plaque clearance in 15 min [10]. However, Lucena-Ferreira et al. observed no difference in *Candida spp.* growth after daily immersion in Polident® for 3 min following nocturnal brushing [13]. In addition, some studies have showed an increase in *Candida spp.* growth after denture cleanser use, which might be relevant to the development of DS [19].

Biofilm formation is an important factor in the pathogenesis of DS, and consists of a complex mixture of fungi, bacteria and desquamated epithelial cells that protects the oral microbiota [9]. The adherence of *C. albicans* to the denture surfaces is the first step to successful colonization and the subsequent pathogenesis [12, 20]. In this study, we found that all denture cleansers prevented biofilm formation, and similar inhibitory effects were seen with Clene®, Polident® and 3% NaHCO_3_ solutions. Freitas-Fernandes et al. also found that Polident® inhibited biofilm formation [16]. Clene® showed good anti-adhesion activity in the early stages, whereas Polident® promoted the adhesion of *C. albicans* and increased the density of cellular layers on the polystyrene substrate. No significant differences were seen between 3% NaHCO_3_, PBS and filter-sterile tap water. Furthermore, *C. albicans* adhesion regressed significantly on PMMA disks immersed for 4 weeks in the different reagents, with Clene®, Polident® and 3% NaHCO_3_ showing higher efficacy compared to PBS and filter-sterile tap water. Hayran et al. also found that Polident® tablets significantly inhibited the proliferation of *C. albicans* on all denture resins. Consistent with previous studies [21, 22], none of the reagents significantly affected the surface morphology and roughness of the PMMA acrylic resin. Furthermore, Polident® in fact reduced the roughness compared to the cleanser/water mixture [22].

Polident® is a mixture of peroxide-based compounds (including potassium kaluic acid and sodium carbonate peroxide) and proteolytic enzymes [23]. The alkaline peroxide-based denture cleansers effervesce in water and mechanically disperse the microbial biofilms. In addition, dissolution of hydrogen peroxide releases oxygen that kills the microbial cells via oxidative damage, whereas the proteolytic enzymes break down the biofilm proteins [24]. Clene® is a mixture of yeast-decomposing, starch-degrading, proteolytic, lipolytic and fibrolytic enzymes, and a peroxide-based compound. There were some minor variations between the efficacy of Clene® and Polident®, which may be attributed to their different composition. The unique composition of Clene® may translate to greater biocompatibility as well, which ought to be explored in future studies.

## Conclusion

Both Clene® and Polident® inhibited fungal adhesion and growth on PMMA acrylic resin base without affecting the surface morphology and roughness. Our findings will have to be validated on DS patients, to observe the therapeutic effect of these reagents in patients.

## Data Availability

The datasets used and/or analyzed during the current study are available from the corresponding author on reasonable request.
